# The economic value of an improved malaria treatment programme in Zambia: results from a contingent valuation survey

**DOI:** 10.1186/1475-2875-4-60

**Published:** 2005-12-15

**Authors:** Felix Masiye, Clas Rehnberg

**Affiliations:** 1 Department of Economics, University of Zambia, P.O. Box 32379, Lusaka, Zambia; 2 Medical Management Centre, Karolinska Institutet, Stockholm, Sweden

## Abstract

**Background:**

Zambia is facing a double crisis of increasing malaria burden and dwindling capacity to deal with the endemic malaria burden. The pursuit of sustainable but equity mechanisms for financing malaria programmes is a subject of crucial policy discussion. This requires that comprehensive accounting of the economic impact of the various malaria programmes. Information on the economic value of programmes is essential in soliciting appropriate funding allocations for malaria control.

**Aims and objectives:**

This paper specifically seeks to elicit a measure of the economic benefits of an improved malaria treatment programme in Zambia. The paper also studies the equity implications in malaria treatment given that demand or malaria treatment is determined by household socio-economic status.

**Methods:**

A contingent valuation survey of about 300 Zambian households was conducted in four districts. Willingness-to-pay (WTP) was elicited for an improved treatment programme for malaria in order to generate a measure of the economic benefits of the programme. The payment card method was used in eliciting WTP bids.

**Findings:**

The study reports that malaria treatment has significant economic benefits to society. The total economic benefits of an improved treatment programme were estimated at an equivalent of US$ 77 million per annum, representing about 1.8% of Zambia's GDP. The study also reports the theoretically anticipated association between WTP and several socio-economic factors. Our income elasticity of demand is positive and similar in magnitude to estimates reported in similar studies. Finally, from an equity standpoint, the constraints imposed by income and socio-economic status are discussed.

## Introduction

Malaria remains the leading cause of morbidity and mortality in Zambia. The Zambian Ministry of Health estimates that the number of malaria cases has tripled in the past three decades to more than 4 million clinical cases and 50,000 deaths per year in a population of less than 11 million people [1]. The socio-economic costs of the malaria burden have produced visible impacts on the livelihoods of poor households [2]. At the macro level, Zambia's public health system is barely coping with the economic burden imposed by malaria. Currently, there is little hope for Zambia reaching the targets outlined in the Roll Back Malaria declaration at Abuja [3]. In fighting malaria, provision of adequate treatment of malaria clinical episodes remains a key challenge for Zambia. However, the recent change in the treatment policy from sulphadoxine-pyrimethamine (SP) to artemesinin-based combination therapy (ACT), presently Coartem^® ^(artemether-lumefantrine) for uncomplicated malaria, has fuelled critical debate about the 'affordability' and 'sustainability' not just of ACT but of the entire malaria control strategy of a poor country like Zambia [4].

The World Health Organization (WHO) and several independent commentators have stated that the chief constraint in the fight against malaria facing many poor countries is inadequate resources [5,6]. The WHO [5] hints that a multi-pronged approach is vital to mobilizing sustainable financing for anti-malarial interventions in Africa. In Zambia, and many other African countries, mobilization of domestic and external resources forms the centrepiece of this campaign, and Zambia, like many other African countries, has been severely hampered by the dearth of knowledge about current expenditures on malaria [5,7]. To achieve desired investments in the fight against malaria, a comprehensive economic accounting system is needed [8].

This paper reports on a methodology based on the economics of eliciting desired financial investments in malaria management interventions in Zambia. The economic approach assumes that society aspires to be both disease-free and economically prosperous. The synergy between growth and malaria burden has also been demonstrated [9,10]. Further, [8] it stresses the relevance of economic measurement: "in trading off between equally deserving demands on health budgets, and more broadly, national development budgets. A systematic demonstration of the scale of the economic impact of an investment in anti-malaria interventions becomes crucial in this debate" [8].

Specifically, the paper applies the willingness-to-pay (WTP) framework for estimating how much should be allocated to malaria treatment (case management) in Zambia. WTP has not been widely applied to estimate the economic benefits of public programmes nor evaluate broad health care programmes in an African context, though it has been applied extensively in environmental and health fields in developed countries.

Several types of policy-relevant information can be generated from WTP results. First, information on community WTP for malaria control is vital for mobilising domestic resources and fostering partnerships which are a centrepiece of the World Health Organization's Roll Back Malaria campaign. Second, by providing the perceived economic benefits of an intervention, WTP forms an input into the cost-benefit analysis (CBA) framework which promotes the important notion of 'value for money' [5,11]. Promoting efficiency through better public spending will make more resources available for malaria. Third, armed with WTP data, malaria programme managers can advocate more effectively for more resources from the Highly-Indebted Poor Country (HIPC) relief to be channelled towards malaria. This economic approach has been proposed by many advocates as one of the viable ways of eliciting benefits and costs of public programmes [8,12].

Finally, WTP can be used to study the socio-economic factors that influence utilization and uptake of malaria treatment services. This is crucial in Zambia given the intimate link between malaria and poverty. Specifically, such analysis is useful for identifying and mitigating the impacts of various socio-economic and demographic barriers to access to malaria interventions among population groups. Similarly, WTP information could be useful in nurturing strategic partnerships with communities which is key to fostering sustainable financing for malaria control [5].

### A theoretical model of household demand for malaria treatment

In this section, a theoretical model of demand for malaria treatment based on the Grossman health production is developed. An underlying premise of this model is that health care commodities are economic goods (i.e. they generate utility and are scarce) and demand for these commodities can be examined within a general household utility maximization framework. Following the benevolent dictator model [13], it is assumed that a household head makes appropriate choices for the entire family. That is, each member of the family directly enters the decision-maker utility function. A household health-related utility function was defined as follows:

*U *= *U*(*H*,*X*)     (1)

Following [14], the utility function contains two arguments good health and other goods, implying that a household derives utility from consuming its health stock (H) and a bundle of other commodities, which is denoted by X. Further, the demand for a malaria treatment is derived from the demand for good health which in turn is inferred from the desire to produce good health. A household produces its health capital by a production function which is typically presented as,

*H *= *H*(*M*,*Z*,β;Ω)     (2)

where *M *is malaria services, *Z *is a composite of goods that impact on health care, β is a vector of exogenous factors, such as environment that can affect health status, and Ω is a vector of household characteristics which include socio-economic status, demographic composition, health status, and so on, which are assumed to impact on household care demand decisions.

From the Grossman model, the accompanying key assumptions include the following; (a) health capital (H) depreciates during an individual's lifetime, (b) an individual has a good assessment of his or her health status at any given point in time, and (c) an individual invests in his/her personal health in order to reduce the rate of health-capital depreciation and to keep his/her health stock at a desired level. Such investment may involve purchasing M. In this model assumes that malaria treatment is presented as one of the means to improve the ability of a household to produce overall health. Hence, the malaria treatment programme is distinguished from 'other' health commodities. Thus, households invest in malaria treatment depending on their own internalized risk of contracting, and falling ill of malaria.

*XP*_*X *_+ *MP*_*M *_+ *ZP*_*Z *_= *Y *    (3)

where X is a composite of all other non-health goods, M is malaria treatment, Z is all other health goods, P are vectors of prices of the corresponding commodities X, M and Z.

Households solve their utility maximization problem to arrive at optimal bundles of X, M and Z subject to their budget. In the context of the malaria programme, the demand functions resulting from the maximization health-related utility can be represented as;

M* = *m*(*P*_*M*_,*P*_*X*_,*P*_*Z*_,*Y*)     (4)

where, Y is income, *P*_*M *_and P_*X *_are the price vectors for malaria treatment commodities and non-health goods respectively. The demand function can then be used to study the effects of this set of variables on the demand for health care.

The maximization solution in equation (4) can be translated into a money metric framework as the minimum cost of buying the bundle M* [15]. In this context, the benefit of a treatment package is the amount of money that a household is willing to forgo from their income in exchange for obtaining guaranteed access to treatment, M* while keeping utility constant. This is represented as,

*WTP *= *V*(*Y*^1^,M*\)-*V*(*Y*^0^,*M *\)     (5)

The intuitive interpretation of the willingness-to-pay approach is the amount individuals are willing to pay to acquire the benefits of a programme that keeps them healthy and alive. In particular, in this model the health effects of M are to reduce the sick days that a household would otherwise suffer if they had no access to treatment for the various illnesses. In this model, the WTP provides an economic value of the malaria treatment programme [16].

Theoretical foundations of the demand for an improved malaria treatment regimen in Zambia can be considered in terms of impact on restoring good health. Apart from prevention, treatment of malaria has important consequences in this utility model. The positive health effects of malaria treatment are demanded for a number of reasons [12]:

• Households wish to maximize the time they stay without malaria for the direct health-utility of being well and also to avoid productivity losses associated with malaria episodes. The time spent away from economic activity due to malaria has greater opportunity costs for the people in low-income countries compared to people in high-income countries. The absence of social security systems and the sensitivity of household current incomes to economic shocks in poor countries increase the sensitivity to malaria [6].

• Malaria, if not treated adequately and timely, can generate catastrophic wealth effects which have been documented to produce a vicious cycle of 'wealth poverty' and 'health poverty'. This is not the case in developed countries where there is generally good access to health insurance which tends to smooth out economic losses.

• Malaria episodes are associated with external economic losses in terms of the opportunity cost of care-givers' time. Externality effects are arguably likely to be stronger in many African societies.

• Treating malaria in malaria-endemic societies produces long-term welfare effects on households by reducing the debilitating effects of malaria on children's mental and physical capabilities.

The implementation of this model is described in the next section.

## Methods

### Survey design: the WTP question design

Respondents were asked to imagine a situation where health services ceased to be offered through a general tax arrangement. Practical illustrations were offered during the interview to help respondents visualize this hypothetical scenario. For example, respondents were informed that the government was proposing to change the institutional arrangement for financing health services in order to enhance effective service delivery. In this proposed model, the institutional framework would be an integrated, community-based health authority. These health authorities would operate health insurance schemes and offer a comprehensive package of health care services designed along disease categories. Enrolment into the scheme required that members pay an amount in the form of a premium for any service they would value.

In this case, the package for malaria was designed to provide an improved and effective malaria treatment programme. Specifically, under this programme households would be provided with full access to diagnostic and treatment services in the event that they need those services. On the WTP question card, used as visual aid in eliciting WTP, specific benefits, included the following range of services were described to respondents: "access to a qualified doctor, nursing care, laboratory and other diagnostic services, access to all necessary anti-malarial medicines and hospitalization costs such as food, laundry and so on, deemed necessary by clinicians and health facility managers". The benefit proposal included setting up an infrastructure (malaria office) for co-ordinating malaria programmes in the district. However, costs not related to institutional care, such as transport, income-loss compensation and cares' costs were not part of the benefit package.

The idea of this question was to simulate a system in which people were guaranteed access to a decent quality of malaria treatment at the point of need. Failure to pay anything meant that the respondent was not going to have access to any malaria treatment option at all. Finally, the survey sought to collect some basic data on households' socio-economic and demographic characteristics. Self-reported income was recorded, though in rural areas the monetary value of liquid assets was included in the estimation of total household income. These assets typically included agricultural outputs, fish, charcoal, goats, chickens and others. No fixed assets such as land, houses, ploughs, bicycles, radios, etc. were included. The main reason for excluding these assets was that it was difficult to value the assets as most of these were old, dilapidated and the owners had no idea of the purchase price. Land, houses and rural huts were also not valued for a lack of suitable market data. This might prove to be a weakness as liquid assets tend to be more variable than assets and may under-estimate household income.

### Study sites and survey administration

The survey was conducted in four districts around Zambia representing two urban (Lusaka and Ndola) and two rural districts (Mazabuka and Kalomo). Malaria is endemic in all these areas. A multi-stage sampling framework was adopted to select subjects from these four districts. Within each districts, two sites were selected from within and outside a radius of 12 kilometres of the central business district area. The 12 kilometres were chosen arbitrarily. Further, within each site (i.e. community), the 5^rd ^to 7^th ^household was picked for inclusion into the survey.

WTP was elicited using an interviewer-administered, structured questionnaire. The researcher with the help of two thoroughly trained enumerators administered this WTP instrument in face-to-face interviews. Only household head or spouses were interviewed. Households were none of these were present were omitted. Preliminary survey administration protocols included soliciting consent and permission from the respondents. The researcher presented an introduction and purpose of the survey. Importantly, once permission was granted, the interviewer assured the respondents that their responses were valuable and would be used to inform policy on funding arrangements for public health care.

A major part of the preliminary part of the interview was devoted to explaining the meaning of WTP in simple terms. Respondents were reminded that their WTP should represent the value they put on such a package of services. At the same time, occasional reminders to respondents about careful consideration of budgets and other household requirements formed an important part of the enumeration process. To facilitate respondent participation and improve validity, all questions were translated into the local language by the enumerator. Finally, careful guidance was generally given to respondents throughout the period of the interview. The survey was conducted from January to May, 2002. Our response rate was 98% giving 274 usable responses.

## Results

### Descriptive statistics of the sample

The sample gives the following summary statistics from the sample compared with population data as published in official Zambian statistics (far right). The data shows that the sample represents the true statistical picture in the population in terms of income, age, sex, household size and literacy levels. However, it can be noticed that the sample under-represents the population living in rural areas, which according to official statistics is around 60 % (Table 1).

**Table 1:** Summary of sample characteristics

	**Sample**	**National**
Size	312	10,800,000
Average age (std dev.)	39.4 (9.79)	37
Household size	6.59 (2.47)	6.5
% living in rural areas	23	62
% Males above basic education	77	15
% Females above basic education	53	9
Average Income (US$)	349.50 (446.64)	**360**

### Statistical estimation of WTP for malaria

The mean WTP for malaria was estimated statistically using interval regression since the data were gathered using payment cards. The regression model below was estimated to give coefficients which were used in estimating mean WTP. In the proceeding section, the determinants of WTP are analysed. A parametric function was used to estimate the mean WTP for grouped data following [17]. According to this approach, the mean of the untransformed dependent variable (i.e. WTP) is estimated using



where,  and  are the expected values (or means) of the significant predictors of WTP (see results below) while the β'*s *are their respective coefficients. σ^2 ^is the variance of the model. The mean WTP equation above calculates the average WTP presented below, which was influenced by at least five explanatory variables identified as reported in the regression results in Table 2 in the index. The mean WTP was multiplied by the population of Zambia in order to derive the aggregate economic value of malaria treatment. This estimate can be interpreted as the economic value of, or a measure of demand for, an ideal malaria treatment programme.

**Table 2:** Household WTP estimates

*Statistic*	*Estimate US $*
Mean WTP	46.67 (ZAM K233,380.02)
Median	35.92 (ZAM K179,624.92)
Aggregate WTP^a^	77,076,666.30
Aggregate WTP^b^	56,794,956.07
Per capita^a^	7.08
Per capita^*b*^	*5.22*
% GDP^a^	1.8

Exchange rate: US$ 1 = ZMK5,000 based on foreign exchange bureau rate that prevailed in much of 2002–3

Notes: superscripts a, b represent per capita WTP based on mean WTP and median WTP respectively.

According to [17], the median is calculated as .

Both the mean WTP and median WTP are presented as recommended in the literature [18]. As can be seen the median is much lower than the mean due to the sensitivity of the mean to skewness in income. However, in further analysis mean WTP is used on the grounds that from a CBA point of view the mean is the theoretically correct statistic to use. The mean, when multiplied by the population size, gives the total or aggregate benefits which have been reported in table 2 for Zambia [18].

The magnitude of WTP for malaria treatment relative to Zambia's gross domestic product per capita suggests that malaria treatment has significant economic value. Despite the common threat of over-valuation in many WTP surveys, these results do appear to be in line with findings from studies in similar socio-economic contexts. A similar study found a WTP of about US$2.00 per capita for *an increase *in households' access to more and better treatment and diagnostic services for malaria in Nepal [19].

The unpublished national health accounts estimates indicate that total health expenditure in Zambia was about K917 billion or approximately US$230 million in 2002 United States dollars. This implies that the desired treatment programme for malaria represents 34% of *current *total health expenditure. However, this ratio would conceivably be substantially less than 34% of desired total health expenditure.

### Socio-economic and demographic determinants of WTP

A regression model was formulated and estimated to capture the determinants of WTP for malaria treatment. Initially, the model included nine explanatory variables and their expected signs (Table 3). To capture income, the survey relied upon self-reported income. Three dummy variables were used to depict the impact of health status on WTP. First, HS1 elicited an overall assessment of the household health status over the preceding six months. The variable HS1 was defined to carry a one if the respondent had poor self-assessed family health status, and zero otherwise. Similarly, the second (HS2) was one if there had been an occurrence of acute short-term illness (a maximum of 21 days of full recovery) in last six months and zero otherwise.

**Table 3:** Determinants of willingness to pay and expected signs

*Variable*	*Expected sign*
Income	+
Education of household head	+
Education of spouse	+
Location (rural or urban)	+/-
Poor Health status	+
Sex of respondent	?
Sex of household head	?
Household size	+/-
Age	+/-

The last dummy (HS3) carried a value of one if the respondent or any member of the family suffers from a chronic illness and zero otherwise. HS3 captured existence of chronic illness which was operationalized as an illness which requires lengthy treatment or regular visitations to a health facility of say once or twice a month continually for at least six months. This typically captured ill-health associated with long term conditions such as diabetes, tuberculosis, stroke, and so on. Of course it can be difficult to distinguish consistently chronic from acute ill-health. The survey relied upon the respondents' own self-assessment of what is chronic and what is not. Local terminologies such as "on and off" were useful during the survey in characterising chronic illness.

The variable Education was assigned a value of one if the head of the household had attained at least grade nine, and zero otherwise. In Zambia attainment of grade nine is conventionally considered to be the benchmark for basic education and functional literacy. Other variables include age of household head, location and sex of household head. Age was a continuous variable while the variables sex of respondent or household head were assigned a value of one for males and zero for females. Location was assigned a value of one if respondent lived in an urban area and zero otherwise.

After taking account of diagnostic problems, particularly multicolinearity, normality and specific errors, the model below was specified. In particular, income and WTP variables were transformed to natural logarithms in order to minimize normality problems and also to avoid heteroscedasticity. The variables location, age of spouse and education of spouse were dropped on account of their correlation with income, age of household head and education of household head, respectively. Further, the robust regression option, which uses the maximum likelihood (ML) estimation procedure, was adopted in order to derive heteroscedasticity-corrected standard errors as well as parameter estimates.

log*WTPMAL *= β_0 _+ β_1 _log*INCOM*_*i *_+ β_2_*EduHead*_*i *_+ β_3_*HS*1_*i *_+ β_4_*HS*2_*i *_+ β_5_*HS*3_*i *_+ β_6_*SEX*_*i *_+ β_7_*HHSiz*_*i *_+ β_8_*AGESQ*_*i *_+ ε_*i*_

The regression results are presented in Table 4. As can be seen the coefficient on the variable income is positive and significant at the 1% level.

**Table 4:** Regression results of determinants of stated WTP

	***Regression Results***	
*Variable*	*Coefficient*	*Robust Std Err.*

Log Income	0.2914	0.04940***
Log AGESQ	-0.0001	0.00008
HS1	0.2908	0.13867**
HS2	0.3184	0.2446**
HS3	0.1600	0.12098
Education head	0.2047	0.12384*
Household Size	0.0349	0.02763
Sex Respondent	-0.0434	0.11194
Constant	4.7380	0.73705**
Sigma	0.7815	0.0331***
Log-likelihood	476.775	
McFadden's R-squared	0.07	
Wald Chi-sq stat	98.88***	
No. of Observations	274	

Notes:

The symbols *, ** and *** denote significance at 10%, 5% and 1% levels respectively.

The estimation results presented in Table 4 generally indicate that the empirical model performed quite well. In particular, measures of overall model significance, namely the F and log likelihood statistics were highly significant, indicating that the specified model would be better than a constant only model. Regrettably, the recorded explanatory power of the model, as given by the McFadden R-squared, was quite low at 7%. This result suggests that the variables included in the empirical model do not explain variations observed in the dependent variable. However, such levels of explanatory power are comparable with those from similar studies [20-24]. Multicollieanity is usually one of the factors blamed for low explanatory power in many survey-based studies.

In terms of the individual explanatory variables, estimation results show that virtually all coefficients generally exhibit expected signs and, in several cases, statistical significance as well: four out of eight coefficients are significant within 0.000 <*p *≤ 0.10 levels of significance. The estimated coefficient on the income variable was positive and highly significant indicating a high income-effect. Everything else being equal, high-income earners are willing to pay substantially more than low income earners. This result suggests that the malaria treatment commodity is a normal good [25]. Given the specification of the WTP model, the coefficients on the income variables can be interpreted as the income-elasticity of demand. The measure of income elasticity of 0.29 reported in this paper suggests that a 10 % increase in household income increases the demand for the malaria treatment by about 3 %. The magnitude of the income effect is reasonably close to those reported in other similar studies [25].

Two of the three health status indicators, HS1 and HS2, were found to be positive and significant at the 5% level. This result suggests that households who reported a generally poor self-assessed health status overall (HS1) were willing to pay more than households who had a good self-reported health status. Then, households who reported an experience of short term ill-health (HS2) within the six months prior to the survey were willing to pay more than those who did not report any such specific illness. This result is rather surprising given that economic theory would suggest that an increasing marginal disutility of illness associated with chronic ill-health should generate a positive and highly significant effect on the WTP. In other words, all else being constant, a household's health status should be negatively related with the marginal utility of seeking insurance. But there are plausible reasons to explain this. One of the reasons for this finding could be that problems of disclosure prevented respondents stating their health status. Another explanation could be that some might not have been aware that their illnesses were chronic at that stage. There was no significant correlation among the three indicators of ill-health.

Sometimes it is suggested that WTP may have a curvilinear relationship age [23]. The rationale is that, everything else being equal, WTP is expected to increase with age up to a point (e.g. during the first fifteen years of life when demand for health care is relatively low), decline through a certain age range (say between 15 and 40), and then increase in the later years as people grow older (of course in Zambia with a life expectancy at barely 40, this may not be the case). So both age and AGESQ (the square of Age) were tested. When both variables, AGE and AGESQ were included, there was noticeable multicollinearity in the estimated model while individually only AGESQ proved to be significant. The variable AGE was dropped in favour of lnAGESQ, which is the logarithm of AGESQ. The sign of the coefficient on the variable AGESQ was as expected positive to signal that age had a generally positive impact on WTP.

In terms of education, the coefficient was also correctly signed and significant. In quantitative terms, the magnitude of the effect of education is illustrated as follows. An interpretation of coefficients on dichotomous variables is provided by [26] which indicates the effect of a variable on the dependent variable by the quantity (exp()-1). Thus, households headed by individuals with more than nine years of education (beyond 9 years is often considered as functional illiteracy) were willing to pay 23 % more for the malaria programme than those headed by less educated individuals. This is a key result established in both the theoretical and empirical illustrations of Grossman-based models that better educated individuals (assumed to possess a higher level of human capital) are more likely to have a higher demand for health insurance programmes.

The sex of the respondent does not appear to have any significant effects on WTP, although it carried a positive sign. It is necessary to point out that the dummy variable for education was assigned a value of one for male and zero for female heads of household respectively. This means that a positive coefficient estimate implies that men are willing to pay more than women. Overall, empirical results in this study display a picture that is in satisfactory conformity with both theoretical predictions and empirical findings in the literature. This gives credence to the valuation technique and results obtained in terms of validity and reliability. Indeed, it would be difficult to imagine that such regularity, in both the WTP estimates and multivariate analysis findings, could be accomplished with a bogus instrument. Therefore, all the acknowledged weaknesses notwithstanding [27-29], these findings can be considered to be reasonably valid and reliable estimates of public demand for these programmes.

## Discussion and conclusion

Policy makers in Zambia and many other African countries are operating in an environment in which reliable information on the social cost and benefit of malaria programmes is virtually absent. This in itself constrains action as, more often then not, policy makers tend to respond to signals raised by such information. In an era afflicted by various communicable diseases, policy makers are desperate to react to signals. Measuring the economic cost of programmes provides one such signal. In this study, the economic benefits of malaria treatment for Zambia have been estimated.

Several possible uses of such information for policy can be outlined. First, the WTP results have provided a scientific, albeit imperfect, estimate of the social value of an improved malaria treatment programme for Zambia. Specifically, such information can be used to stimulate awareness about the potential benefits of malaria treatment. WTP can be used to provide social values of various other treatment programmes which would then be used to rank public interventions in some 'order of importance'. Overall, such a methodology could help in the evaluation of alternative public programmes.

Second, WTP information can be used as an input into cost-benefit-type policy decisions. In this case, policy makers can compare the net benefits, defined as the WTP minus the financial cost of providing malaria treatment. The rule is that the programme should be considered if the benefits outweigh the costs, that is, if the net benefits are positive. In terms of ranking, the programme with the highest net benefit is most preferred. It would be interesting to compare malaria treatment with other malaria interventions such as prevention, or indeed with other disease programmes. Such analyses are intended to maximise impact, as measured by the monetary social benefit in WTP, from available scarce resources.

Third, the study findings also indicate that considerable inequalities among population groups in demand are evident in the WTP results, with demand being positively affected by income. For instance, this can be used to serve some important equity concerns such as instituting policy measures that boost demand among specific groups especially in the context of low and unequal distribution of access to care. Understanding determinants of WTP is vital for policy strategies aimed at boosting demand and expanding coverage fort malaria interventions. Similarly, such information is also crucial for designing long-term sustainability frameworks for malaria management and control in Zambia. The results of this study have raised concerns for both equity and programme sustainability.

Finally, a more general use of WTP and cost-benefit analysis (CBA) is that it entrenches systematic and evidence-based decision-making. Competition for resources among managers of major disease programmes, with each insisting that their burden is higher than society or policy-makers seem to understand, requires that some defensible ways of interpreting either public demand or resource needs. WTP, though not the only possible means of estimating social value, provides an option. In real life, decision-makers are often caught between vocal demands from various stakeholder groups. For example, beneficiaries of treatment for cardiovascular diseases, many cancers, and more recently HIV/AIDS tend to form relatively strong interest groups (partly because they tend to be economically and/or socially powerful) and project a strong voice.

On the contrary, sufferers of the bulk of infectious and other so-called 'diseases of poverty', such as tuberculosis, malaria, malnutrition, maternal conditions, etc., tend to disproportionately represent the economically and socially vulnerable, politically dissipated, and the least powerful in terms of social advocacy. In principle, such situations pose the danger that decision makers may devote an imbalance of resources towards big benefits to small identified groups as opposed to benefits to wider groups. Decision makers can benefit from a rational and 'evidence-based' method for weighing up alternative ways of spending resources, even if WTP or CBA are unlikely to be the only frameworks for doing so.

## Authors' contributions

Felix Masiye formulated the research problem, developed the methodology including survey instruments, collected the data, participated in the analysis and writing of this manuscript. Clas Rehnberg critically reviewed the methodology and participated in drafting this manuscript.
